# Prevalence of soil-transmitted helminthes and its association with water, sanitation, hygiene among schoolchildren and barriers for schools level prevention in technology villages of Hawassa University: Mixed design

**DOI:** 10.1371/journal.pone.0239557

**Published:** 2020-09-24

**Authors:** Wondwosen Abera Gitore, Musa Mohammed Ali, Amanuel Yoseph, Adane Ermias Mangesha, Alemu Tamiso Debiso

**Affiliations:** 1 School of Medical Laboratory Science, College of Medicine and Health Sciences, Hawassa University, Hawassa, Ethiopia; 2 School of Public Health, College of Medicine and Health Sciences, Hawassa University, Hawassa, Ethiopia; 3 Department of Environmental Health, College of Medicine and Health Sciences, Hawassa University, Hawassa, Ethiopia; Ministry of Health and Sports, MYANMAR

## Abstract

**Background:**

Soil-transmitted helminths (STH) remain one of the most common causes of morbidity among children in Ethiopia. Assessment of the magnitude of STH and its association with water, sanitation, and hygiene (WASH) and identify barriers for school-level prevention assist public health planners to prioritize promotion strategies and is a basic step for intervention. However, there is a lack of evidence on the prevalence of STH and its association with WASH and barriers for school-level prevention among schoolchildren.

**Objective:**

To assess the prevalence of STH and its association with WASH and identify barriers for school level prevention in technology village of Hawassa University; 2019.

**Methods:**

An institution-based analytical cross-sectional study was conducted on a sample of 1080 schoolchildren from September 5 to October 15, 2019. A two-stage cluster and purposive sampling technique were used to draw the study participants. A pretested, structured questionnaire, observation checklist, and in-depth interview were used to collect the data. Two grams of stool samples were collected from each study participant and examined using direct wet mount and Kato-Katz technique. Data were entered into Epi Info version 7 and analyzed using SPSS version 25. Both bi-variable and multivariable logistic regression analyses were done. Qualitative data were analyzed using thematic content analysis method by Atlas-Ti software and presented in narratives.

**Results:**

The overall prevalence of STHs was 23.1% (95% CI = 21.4, 27.6). The identified predictors of STHs were large family size (AOR = 2.03; 95% CI = 1.53–3.99), absence of separate toilet room for male and female (AOR = 3.33; 95% CI = 1.91–5.79), toilet not easy to clean (AOR = 2.17; 95% CI = 1.44–3.33), inadequate knowledge about STHs (AOR = 2.08; 95% CI = 1.07–3.44) and children who had travelled greater than 100 meters to access toilet (AOR = 3.45; 95% CI = 2.24–8.92). These results were supported by the individual, institutional, socio-economic and cultural qualitative results.

**Conclusion:**

The STHs was moderate public health concerns. Reinforcing the existing fragile water, sanitation and hygiene programs and regular deworming of schoolchildren may support to reduce the burden of STHs. Also, increasing modern family planning methods utilization to decrease family size is recommended.

## Background

Soil-Transmitted Helminths (STHs) is a name given for groups of helminthes caused by parasitic worms. The most common STHs are *Ascaris lumbricoides*, *Trichuris trichiura*, *Strogyloides Stercoralis*, and hookworms (*Necator americanus* and *Ancylostoma duodenale*) [1]. They can cause diarrhea and under-nutrition among the different segments of the population. Children and marginalized communities are the most susceptible categories since they have more probability of contact with contaminated soil in their daily activities [2].

Lack of clean water supply, poor personal and environmental hygiene and sanitation are the major causes of infection with STHs. STHs are common in contaminated, heavily populated, and areas with minimum hygiene [3]. Infection with STHs is manifested by nausea, vomiting, diarrhea, abdominal pain, blood and protein loss, rectal prolapse, and weight loss. These manifestations may be more devastating among schoolchildren, malnourished, and immune-compromised individuals than general population groups [4–6]. Diarrhea is the main complication of soil-transmitted helminths and the first top cause of morbidity and mortality of young children in the outpatient department in less developed countries [7, 8]. In addition, infection with STHs can increase micronutrient deficiencies, protein-energy malnutrition, and iron deficiency anemia [9]. Moreover, STHs have been related with long term consequences on school age-children these include physical and cognitive growth retardation, school absenteeism, poor academic performance, and stunted growth [10, 11].

Many studies have related STHs infection with low socio-economic status, poor health-seeking behavior, low availability of health service, food insecurity, and low deworming uptake. However, almost all of these studies focused on preschool children who have close follow up and special care given by parents/caregivers [12–15]. Furthermore, these studies also did not assess the effect of WASH on the prevalence of STHs among schoolchildren.

Schoolchildren are the most susceptible groups at risk of STHs due to their practice of walking and playing barefoot, poor nutritional status and knowledge on the transmission course of the parasites, insufficient healthcare, and poor personal hygiene [2, 6]. Therefore, they need close follow up and special care. In the presence of many prevention and control strategies such as mass drug administration and WASH, STHs are a widespread health problem in developing countries [1].

The federal ministry of Ethiopia developed National Master Plan to eliminate STH by 2020 [16]. However, a study conducted in Babile town, Eastern Ethiopia reported a 27.2% prevalence of intestinal helminths. [17]. *A*. *lumbricoides* and *T*. *trichiura* are the commonest and widely distributed parasites throughout Ethiopia [18]. According to the Sidama zone health department 2015 report, the prevalence of STH in the technology village was 45%. It has far above the national target (less than 1%) and uniformly affects almost all Kebeles of the village. Schoolchildren shared for 30% of the overall 45% STH prevalence in the technology village [19]. In the technology village of Hawassa University; deworming treatment coverage among School-age children was 60% for STH, which was far below the zonal target of 90% in 2018 [20].

There was a high transmission rate of STHs in the village despite of preventive and curative actions undergoing. Among these, de-worming of all schoolchildren using Albendazole or Mebendazole were conducted 2 times per year regularly. The most common type of toilet facility in the village was a pit latrine without a slab or open pit [19].

Although to prevent and control STHs infection, the World Health Organization (WHO) recommended a school based deworming program with a WASH component. However, there is limited evidence on the prevalence of STHs and its association with WASH among schoolchildren in the technology village of Hawassa University. Furthermore, assessing the current prevalence of STHs and identifying the predictors of STHs infection is vital to guide implementers, public health planners, stakeholders, and policymakers to plan and design precise intervention strategies to eliminate STHs. Therefore, this study aimed to assess the prevalence of STH and its association with WASH and to explore barriers for schools level prevention strategies among schoolchildren in the technology village of Hawassa University, Southern Ethiopia.

## Methods and materials

### Study area

This study was carried out in technology village of Hawassa University, Southern Ethiopia. The village is found 310 km from Addis Ababa, the capital of the Ethiopia. It has consisted of 6 districts and 168 kebeles (the least administrative unit of Ethiopia with an estimated 1,000 households). According to the 2019 central statistical agency report of Ethiopia, the total population of the village was estimated to be 636, 146 (3% urban and 97% rural). The overall potential physical health service coverage of the village by public health facilities was 85%. The village has one general and six district hospitals, 52 health centers, 213 health posts and 12 private medium clinics and 14 private drug stores [19]. According to the agro-ecological region, about 20%, 30%, and 50% of the population reside in the lowlands (<1750 m above sea level (ASL)), midlands (1,750–2,300 m ASL) and highlands (>2,300 m ASL), respectively. According to the seasons of year classification, the temperature of village varies from 16 to 38°C and the average rainfall is 1100 mm per annum. The most common rainy seasons is extended from late July to early October. The farming is the main source of income-generating activity (for about 85% of the population) in the village. The most common crops grown in the village are maize, coffee, enset *(Enset ventricosum)*, khat, cereals, and barely [21]. Poor hygiene and sanitation related illnesses, like acute watery and bloody diarrhea, trachoma, typhoid fever and intestinal helminthic infections are among the important causes of morbidity and mortality in the village [19].

### Study design and population

An institution-based analytic cross-sectional study was done using quantitative and qualitative approaches in the technology village of Hawassa University from September 5 to October 15, 2019. The source and study population for the study were all schoolchildren and randomly selected schoolchildren, respectively. Children who had serious diseases received anti-helminthic treatment within the last one month before the survey, diarrhea at the time of stool sample collection, and absent from School for 3 consecutive visits were excluded from this study.

### Sample size determination

The minimum needed sample size was calculated using Epi-Info^™^ version 7. The sample size required to estimate the prevalence of STHs was calculated assuming the anticipated prevalence of STHs to be 54.9% based on a study among schoolchildren in Northwestern Ethiopia [22], margin of error (d) of 0.05, z-value of 1.96 for a 95% confidence level, design effect of 2 and a 10% compensation for nonresponse rate. Hence, the final calculated sample size was 836. The sample size required to identify the predictors of STHs was estimated considering variables significantly associated with STHs in previous similar studies [22–24] and setting the level of confidence at 95%, power at 80%, ratio of unexposed-to-exposed at 1:2 and expected nonresponse rate at 10%. In the study among schoolchildren in North Western Tigray regional state, Ethiopia [23], the prevalence of STHs among schoolchildren who were users of latrine for defecation (unexposed) was 14% and open field use for defecation was associated with STHs with an AOR of 2.5. In the study in Durbete town, Northwestern Ethiopia [22], the prevalence of STHs among schoolchildren who were washed their hands before eating a meal always (unexposed) was 11% and habit of never washing hands before eating a meal was associated with STHs with an AOR of 3.0. Further, in a study among schoolchildren of rural Tebre Tabor, North West Ethiopia [24], the prevalence of STHs among schoolchildren who were trimmed fingers (unexposed) was 13.2% and untrimmed fingers was associated with STHs with an AOR of 3.2. Based on above inputs, the final calculated sample sizes were 548, 1199 and 513. Therefore, the sample size of 1199 was used as the minimum required sample size for the current study as it would be adequate to address all objectives of this study. The sample size for qualitative study was estimated based on the recommendation of Creswell and Morse for Phenomenological studies (5–25 study participants) [25]. Accordingly, we decided to include 20 study participants as per their recommendation. However, we attained level of information saturation after interviewed 15 study participants. We conducted 3 extra in-depth interviews in ordered to assure the real level of information saturation. Therefore, we included 18 study participants to explore the barriers for schools level prevention of STHs.

### Sampling procedure

The calculated total sample size was allocated proportionally between the three agro-ecologic regions (lowland, midland, and highland). A two-stage cluster sampling method was employed to choose schools and students in each agro-ecologic region. In the first stage, 6 schools (three each from rural and urban areas) were randomly drawn from a list of schools developed by the Sidama regional state education bureau for the year 2019. The choice of 6 schools was according to the inclusion of 12 students each from grade three to eight directing to an enrollment of 72 students overall to represent each chosen school. The second stage was used to select Schoolchildren from each school. A random number table in SPSS version 25 was utilized in selecting both the schools and the Schoolchildren within each grade. If students were absent from the Schools for three consecutive visits or under exclusion criteria and there were no other alternatives, the student in the subsequent random number table list was enrolled. Moreover, the consideration to involve 6 schools from each region was also according to the feasibility of logistics and the availability of the budget for the study. Therefore, 18 schools were involved in 3 regions for the study. A purposive sampling technique was used to select study participants for the in-depth interview by considering variability in sex, age, educational status, roles, and responsibility in the Schools.

### Operational definition

#### Seriously sick child

Is a child who apparently looks sick and is unable to communicate and is unusually quiet and inactive.

### Study variables

The outcome variable was the prevalence of STHs which was defined as direct microscopic evidence of one or more helminths during the examination. The independent variables were socio-demographic characteristics such as age, sex, religion, ethnicity, place of residence, family/guardian educational status, family/guardian occupation, family/guardian wealth status, and social media availability; School environmental factors such as school water source, toilet availability, toilet distance from the classroom, separate toilet for male and female, toilet cleaning mechanisms; behavioral factors such as hand washing practice before eating a meal, handwashing practice after using the toilet, use of soap for handwashing, a habit of consuming raw vegetables and fruits, the practice of shoe wearing, a habit of nail biting, and fingernail status (trimming and untrimmed); knowledge on prevention of STH; attitude towards the cause of STH.

### Data collection tools and procedures

The data were collected using a structured interviewer-administered questionnaire, observation checklist, and in-depth interview (IDI) in Sidama language (the local language of the study setting). The questionnaire (study tool) was prepared originally in English, translated into Sidama language and then retranslated back to English. The comparison was conducted to check consistency and accuracy between the two versions of study tools. The data collectors and supervisors were trained on the study tool, interview methods, objectives of the study, and stool sample collection methods by the principal investigator for two days. After the training, the study tool was pre-tested on 5% of a total calculated sample size and 3 IDI was conducted on Schoolchildren in School which was not included in the actual study setting. At that moment, any inconsistency and non-accuracy identified were rearranged accordingly. Data collection was administered by 20 diploma nurses, 4 laboratory technologists, and 12 community health workers. Four health officers prudently supervised the data collection method. The qualitative data were collected using IDI until information saturation was reached. IDI guideline was used to explore ideas of school directors and teachers. The two experienced public health expert with MPH were recruited for note-taking, tape recording, and facilitating the IDI. This information was triangulated with the quantitative technique.

A structured questionnaire comprised four parts. The first parts included socio-demographic and economic characteristics of the study respondents as aforementioned above in the study variables part. The second part comprised 10 questions about the knowledge of STHs. Knowledge questions were both in the form of a multiple responses or the arrangement of yes or no (K1-K10). Correct responses had assigned 1 score, whereas incorrect responses were assigned 0 scores. Finally, the overall knowledge point extended from 1 to 10. The study participants who had scored 50% and below were classified as having poor knowledge, 51% to 74% as having moderate knowledge, and above 75% as scoring high knowledge of STHs.

The third part assessed the attitude concerning STHs. To assess the attitude of the Schoolchildren towards the STHs, 11 questions were tested (A1-A11). The responding and scoring systems were the same to the second part which was about knowledge (correct = 1, incorrect = 0). The overall attitude point extended from 1 to 11. A score of below mean, mean, and above was categorized as a negative and positive attitude towards STHs, respectively. The study questionnaire is now provided as supporting information (S1 File).

The last part included 8 questions regarding the practice of STH. Concerning the Schoolchildren practice towards the STHs, 8 questions were tested (P1-P8), with the same grading system as earlier (correct = 1, incorrect = 0). Overall scores of below mean, mean, and above were categorized as a poor and good practice towards STHs prevention, respectively.

### Stool sample collection and examination technique

About 2 grams fresh stool samples were collected on the spot using clean, tightly corked, leak-proof containers from each Schoolchild. The microscopic examination of stool samples was done using the direct wet mount and Kato-Katz technique to identify the STH eggs or larvae. Two Kato slides per stool sample were prepared using a fixed quantity of sieved 41.7mg of stool on a punched template. The intensity of infection was estimated by averaging the number of eggs per gram (epg) of feces on both slides. Laboratory personnel read each prepared slides. Ten percent of randomly selected stool samples were re-examined by a reference laboratory personnel for quality control. Accordingly, they reported similar species of STHs (no evidence of inconsistent findings). Each reagent was checked for the expired date before conducting a stool sample examination.

### Data processing and analysis

The data were cleaned, coded, and entered into Epi data version 3.1 and all analyses were done using SPSS version 25. All the required variables computations, recoding, categorizations, and calculations were conducted before the major analyses. The descriptive statistical analyses were carried out to get descriptive measures for the socio-demographic and other essential variables of interest. The chi-square(X^2^) test was applied to explain the overall association among exposure and outcome variables. The cross-tabulation was employed to check the important assumption of the chi-square test. A sensitivity analysis was done to examine the impact of missing data using multiple imputation techniques.

The data were analyzed using both a bi-variable and multivariable binary logistic regression model. Those variables with p-values < 0.25 on the bi-variable analysis model were entered into a multivariable binary logistic regression model to find out factors independently associated with STHs adjusting for other factors in the model. The candidate variables were entered into the multivariable binary logistic regression model using the backward (Wald) stepwise regression method. The basic assumptions of the binary logistic regression model like the absence of outliers, multicollinearity, and interaction between independent variables were verified to be fulfilled. The effect modification was assessed by putting interaction terms into the multivariable logistic regression model one at a moment. As a result, all of the interaction terms were statistically insignificant suggesting a lack of significant effect modification between variables of interest. The multicollinearity between the explanatory variables was also considered using a multiple linear regression model. For all variables of interest as the variance inflation factor (VIF) was less than 10 and the tolerance statistics > 1 which shows no evidence of multicollinearity among independent variables. Fitness of the binary logistic regression model was also measured using the Hosmer-Lemeshow statistic, the pseudo-R2, and classification accuracy of the model. The presence and strength of association between STHs and the exposure variables were estimated using adjusted odds ratios (AORs) with a 95% CIs. The statistically significant association between the variables of interest was assured when the 95% CI of the AORs did not comprise 1.

The qualitative data were transcribed verbatim from original Sidama language into the English language and presented in narratives using the study participant’s own words. The texts were read and checked 2 times by the expert transcribers for verification purposes. Qualitative data were coded and analyzed using a thematic content analysis technique by Atlas-Ti software.

### Ethical consideration

The ethical clearance was received from the Institutional Review Board of Hawassa University at the College of Medicine and Health Sciences before beginning data collection with a reference number of IRB/149/10. An authorized letter of approval was obtained from the School of Public Health to the respective Schools. The informed written approval was also received from Schools. The informed written consent was also taken from all parents/legal guardians after clarifying the significance of the study, aims, risks or benefits, rights, privacy, nature of the study, and the range of their participation in this study through students. Finally, the assent from students was obtained before the data collection. Schoolchildren with heavy intensity infections and protozoa parasites were referred to nearby public health institutions for further investigation and treatment. The ethical considerations were addressed by treating those who were diagnosed with STHs using albendazole 400 mg.

## Result

### Socio-demographic characteristics of schoolchildren

From a total of 1199 sample size estimated, only 1080 SAC responded questions, making a response rate of 90.7%. The mean (±standard deviation [SD]) age of children was 118±49 months. The median family size of each studied household was 6 persons. This study revealed that almost all of the study participants were from the Sidama ethnic group and followers of protestant Christianity, respectively. More than 90% of the mothers and fathers had never attended formal education (Table 1).

**Table 1:** Socio-demographic characteristics of study participants in technology village of Hawassa University, Southern Ethiopia, 2019 (N = 1080).

Variables	N (%)
**Age in months**	
84–120	551 (51)
121–168	529 (49)
**Religion**	
Protestant	1080 (100)
**Ethnicity**	
Sidama	1080 (100)
**Mother’s occupation**	
Housewife	992 (91.9)
Farmer	18 (1.7)
Merchant	70 (6.5)
**Father’s occupation**	
Farmer	954 (88.3)
Merchant	79 (7.3)
Government employee	47 (4.4)
**Mother’s educational status**	
Illiterate	1013 (93.8)
Read and write	47 (4.4)
Formal education	20 (1.9)
**Father’s educational status**	
Illiterate	980 (90.7)
Read and write	61 (5.6)
Formal education	39 (3.6)
**Family size**	
Small	352 (32.6)
Medium	335 (31.0)
Large	393 (36.4)
**Grade level of students**	
3^rd^ to 5^th^	540 (50)
6^th^ to 8^th^	540 (50)

### Water supply and sanitation

Improved drinking water supply was only available for 1 (5%) school. None of the schools had any alternative water source like surface water or ground water. A pit latrine was available in all schools and none of the schools had pour water or water flushed toilets. Schoolchildren had traveled more than 100 meters to access toilet in study area. Ratio of students per toilet is greater than 1:200 in 14 (76.9%) of the schools.

### Prevalence of soil-transmitted helminthes

The overall prevalence of STHs was 23.1% (95% CI = 21.4–27.6%). Skin-penetrating STH (*S*. *stercoralis* and hookworms (*N*. *americanus* and *A*. *duodenale*) and orally-ingested STH (*A*. *lumbricoides* and *T*. *trichiura)* were identified in 6.8% and16.34% of schoolchildren, respectively. *A*. *lumbricoides* was the most common infection 121 (11.2%) followed by *T*. *trichiura* 49 (4.5%), *S*. *stercoralis* 41 (3.8%), and hookworm 29 (2.7%). Infection with more than one STH (concomitant infections) was 10 (0.9%) among schoolchildren in the current study. Among those, 4 (0.37%) were demonstrated triple infections by *A*. *lumbricoides*, *T*. *trichuira*, and hookworm. Heavy infection with any STH species was identified in 1.8% of the schoolchildren (Table 2).

**Table 2:** Prevalence of STH among schoolchildren in technology village of Hawassa University, Southern Ethiopia, 2019 (N = 1080).

Variables	N (%)
**Types of STH infections**	
Single	240 (22.2)
Mixed	10 (0.9)
All types	250 (23.1)
**Types of STHs**	
*A*. *lumbricoides*	121 (11.2)
*T*. *trichuria*	49 (4.5)
*S*. *stercoralis*	41 (3.8)
Hookworm	29 (2.7)
**Concomitant infection**	10
Double	6 (0.53%)
Triple	4 (0.37%)
**Intensity of infection**	n = 250
Light	176 (16.3)
Moderate	55 (5.0)
Heavy	19 (1.8)

### Knowledge, attitude and practice of schoolchildren towards STH

The majority of study participants knew that cause of STHs are parasitic worms, symptoms such as diarrhea and abdominal pain, loss of appetite, general malaise, and weakness and individuals with certain conditions are at a higher risk for STH infections (53.0%, 64.3%, and 56.7%, respectively). Similarly, the majority of study participants knew that STHs can be prevented through different mechanisms, contaminated soil, and water, consumption of raw vegetables and fruits are ways of transmission of STHs and treatment were available in the market (63.4%, 60.8%, and 72.3%, respectively). According to our results, the majority, 576 (53.3%) of the Schoolchildren had good knowledge about the STHs. The mean knowledge score was 6.99, indicating an overall 69.9% correct rate for knowledge questions.

The majority, 909 (84.2%) and 641 (59.4%) of the study participants had to think STHs infection can be cured and at risk for STHs, respectively. Less than half of the study participants had confidence that the Ethiopia government was able to eliminate the STHs, while 52.8% had no such confidence. Only 54.0% of the study participants had reported that STHs implies academic performance. The mean (± standard deviation [SD]) correct response score of the 11 questions about the attitude towards STHs rate was 6.4 (± 3.8), indicating an overall 58.2% correct rate on this attitude test. The majority, 569 (52.7%) of the study participants had a negative attitude towards STHs.

The majority of study participants had washed hands with soap before the meal, and after toilet, avoided open field defecation, and wore shoes to prevent STHs infection (61.8%, 82.6%, and 55.3%, respectively). The mean (± standard deviation [SD]) correct response score of the 8 questions concerning the rate of the STHs practice was 4.0 (± 2.7), indicating an overall 50% correct rate in the practices test. The majority, 557 (51.6%) of study participants had poor practice towards STHs (Table 3).

**Table 3:** Knowledge, attitude and practice of study participants toward STHs in technology village of Hawassa University, Southern Ethiopia, 2019 (N = 1080).

Item (correct response)	N (%)
**Knowledge part**	
Do you know the STHs (yes)	819 (75.8)
Do you received a deworming pill in the preceding six months of survey (yes)	748 (69.3)
Cause of STHs is parasitic worm infection (yes)	572 (53.0)
Is STHs a transmissible disease (yes)	771 (71.4)
Contaminated soil and water, consumption of raw vegetables and fruits are ways of transmission of STHs (yes)	657 (60.8)
Diarrhea and abdominal pain, loss of appetite, general malaise and weakness are symptom of STHs (yes)	694 (64.3)
Is there treatment for STHs (yes)	781 (72.3)
Can STHs be prevented (yes)	685 (63.4)
Know the correct prevention methods of STHs (yes)	663 (61.4)
Preschool children, school age children, pregnant and lactating women are at a higher risk for STHs (yes)	612 (56.7)
Knowledge toward STHs (mean ± SD)	(6.99 ± 3.3)
Knowledge toward STHs	
Poor	338 (40.0)
Moderate	116 (10.7)
Good	576 (53.3)
**Attitude part**	
Do you think STHs harm Schoolchildren (yes)	439 (40.6)
Do you think yourself at risk (yes)	641 (59.4)
If you take precautions, can the STH infection be prevented (yes)	731 (67.7)
If you know that raw vegetables and fruits are sources for the transmission of STHs, would you consume raw vegetables and fruits (no)	520 (48.1)
Do you think can STHs infection be cured (yes)	909 (84.2)
Is the available information about the STHs in School sufficient (yes)	650 (60.2)
Are the elimination measures sufficient for prevention of STHs (yes)	671 (62.1)
Is there negative effect of STHs on academic performance of students (yes)	583 (54.0)
Do you think the Ethiopian government able to eliminate the STHs (yes)	510 (47.2)
If you have one of the symptoms of the STHs do you go to the health facility (yes)	485 (44.9)
Do you think inappropriate latrine utilization can cause STH infection? (yes)	706 (65.4)
Attitude towards STHs (mean ± SD)	(6.4 ± 3.8)
Negative	569 (52.7)
Positive	511 (47.3)
**Practice part**	
I wash hands with soap before meal and after toilet (yes)	667 (61.8)
I avoid touching the mouth with unwashed hands (yes)	358 (33.1)
I avoid consuming raw vegetables and fruits (yes)	379 (35.1)
I wear shoes (yes)	597 (55.3)
I avoid playing with soil (yes)	390 (36.1)
I avoid open field defecation (yes)	892 (82.6)
I receive deworming tablet (yes)	696 (64.4)
I keep my fingers clean by cutting nails (yes)	444 (41.1)
Practice towards STHs (mean ± SD)	(4 ± 2.7)
Poor	557 (51.6)
Good	523 (48.4)

### Predictors of STH infections

Results of the bi-variable and multivariable binary logistic regression analyses of STHs are presented in Table 4. Both bi-variable and multivariable binary logistic regression analyses of predictors showed that odds of STHs were 2.03 times increased in schoolchildren living in large family size as compared to those living in small family size (AOR = 2.03; 95% CI = 1.53–3.99; P = 0.028). The absence of separate toilet room for male and female in School (AOR = 3.33; 95% CI = 1.91–5.79; P = 0.001), toilet not easy to clean (AOR = 2.17; 95% CI = 1.44–3.33; P = 0.001) and inadequate knowledge of schoolchildren about STHs (AOR = 2.08; 95% CI = 1.07–3.44; P = 0.001) were positively associated with STHs. Besides, the odds of STHs were 3.82 times increased for children who had never washed their hands before the meal (AOR = 3.82, 95 CI = 1.95–9.88) as compared to those who had always washed their hands. Moreover, multivariable analysis of predictors revealed that odds of being infected with STHs were 3.45 times increased for children who had traveled greater than 100 meters to access the toilet (AOR = 3.45; 95% CI = 2.24–8.92; P = 0.013) as compared to those who traveled 1–30 meters.

**Table 4:** Bi-variable and multivariable binary logistic regression analyses of predictors of STHs among schoolchildren in technology village of Hawassa University, Southern Ethiopia, 2019.

Variables	STHs		COR	AOR
	**Yes**	**No**		
**Maternal education status**				
Illiterate	226 (22.3)	787 (77.7)	0.87 (0.28, 2.63)	0.66 (0.20, 2.23)
Read and write only	20 (42.6)	27 (54.4)	0.33 (0.09, 1.16)	0.23 (0.04, 1.01)
Formal education	4 (20.0)	16 (80.0)	1	1
**Maternal occupation status**				
Housewife	220 (22.2)	772 (77.8)	1.83 (1.09, 3.06)	1.50 (0.82, 2.76)
Farmer	6 (33.3)	12 (66.7)	1.04 (0.34, 3.12)	0.65 (0.17, 2.44)
Merchant	24 (34.3)	46 (65.7)	1	1
**Paternal occupation status**				
Farmer	215 (22.5)	739 (77.5)	1.77 (0.95, 3.30)	1.05 (0.51, 2.15)
Merchant	19 (24.1)	60 (75.9)	1.63 (0.73, 3.60)	1.81 (0.75, 4.38)
Government employee	16 (34.0)	31 (66.0)	1	1
**School water source**				
Yes	21 (36.8)	36 (63.2)	1	1
No	229 (22.4)	794 (77.6)	2.02 (1.16, 3.53)	1.37 (0.07, 2.68)
**Family size**				
Small	23 (19.3)	96 (80.7)	1	1
Medium	54 (12.1)	391 (87.9)	1.73 (1.01, 2.96)	1.75 (0.96, 3.16)
Large	173 (33.5)	343 (66.5)	3.43 (1.29, 4.76)	2.03 (1.53, 3.99)*
**How often wash hands before meal**				
Always	50 (18.3)	223 (81.7)	1	1
Usually	46 (17.8)	213 (82.2)	1.04 (0.67, 1.61)	1.16 (0.71, 1.91)
Sometimes	123 (24.7)	374 (75.3)	1.47 (1.02, 2.12)	0.73 (0.48, 1.10)
Never	31 (60.8)	20 (39.2)	6.89(3.65, 13.15)	3.82 (1.95, 9.88)**
**Toilet distance from class room**				
1–30 m	8 (7.6)	97 (92.4)	1	1`
31–70 m	7 (7.1)	92 (92.9)	1.08 (0.37, 3.11)	0.70 (0.21, 2.26)
Greater than 100 m	235 (26.8)	641 (73.2)	4.44 (2.12, 9.25)	3.45 (2.24, 8.92)*
**Separate toilet room for male and female**				
Yes	232 (27.2)	621 (72.8)	1	1
No	18 (7.9)	209 (92.1)	4.33 (2.62, 7.18)	3.33 (1.91, 5.79)**
**Toilet easy to clean**				
Yes	51 (14.6)	298 (85.4)	1	1
No	199 (27.2)	532 (72.8)	2.19 (1.56, 3.07)	2.17 (1.44, 3.33)**
**Deworming status in the preceding six months of survey**				
Yes	172 (22.6)	588 (77.4)	0.90 (0.66, 1.23)	0.85 (0.59, 1.24)
No	78 (24.4)	242 (75.6)	1	1
**Knowledge**				
Inadequate	126 (33.9)	246 (66.1)	2.41 (1.80, 3.22)	2.08 (1.07–3.44)*
Adequate	124 (17.5)	584 (82.5)	1	1
**Attitude**				
Negative	110 (19.3)	459 (80.7)	0.63 (0.47, 0.84)	0.88 (0.56, 1.37)
Positive	140 (27.4)	371 (72.6)	1	1
**Practice**				
Poor	97 (17.4)	460 (82.6)	0.51 (0.38, 0.68)	0.90 (0.54, 1.50)
Good	153 (29.3)	370 (70.7)	1	1

1: Indicates the reference categories

*: Indicates significant association (p-value < 0.05)

**: Indicate the highly significant association (p-value <0.01).

### Barriers for prevention of STH in school

#### Themes from the interview data

A total of 20 individuals were selected for an in-depth interview. However, after the 15th interview, no new themes were generated from the interviews. Therefore, it was deemed that the data collection had reached a saturation point. We continued data collection for three more interviews to ensure and confirm that there are no new themes emerging.

After analyzing the interview data, two themes emerged which were discussed in this section. These themes were: lack of WASH facilities and poor knowledge, attitude and practice of schoolchildren towards the prevention, and control of STH.

Each of the school headmasters, teachers and community health workers believe that the barriers for prevention of STH at the school level are lack of clean water supply for drinking, and hygienic purposes although few schools provide spring water. Moreover, the lack of toilets with superstructures and separate rooms for male and female students had been a discouraging factor for the appropriate use of toilet facilities. In the interview, the data reveal that the way some toilets are constructed made it difficult for cleaning and managing sludge. The community health workers suggested that the lack of a well-organized school health club was also one of the challenges in preventing STH infection.

The interview data also showed that the school children do not have sufficient knowledge about the mode of transmission and prevention methods of STH. It was also proved through interviews that the school children knew *A*. *lumbricoides* as the only STH. Hookworm infections, *T*. *trichuira* and *S*. *stercoralis* were not recognized by the children. Most school children said, “STHs are transmitted through infectious droplets and transmission has nothing to do with personal or environmental hygiene”. These comments seem to provide evidence that school children do not have the right practice of personal hygiene for preventing STHs.

## Discussion

An institution-based analytical cross-sectional study was conducted to determine the prevalence of STHs and its association with WASH among Schoolchildren and barriers for School level prevention using mixed methods among Schoolchildren in the technology village of Hawassa University, Southern Ethiopia. The prevalence of STHs among the Schoolchildren in the technology village of Hawassa University was 23.1%. Large family size, inadequate knowledge about the STHs, toilet not easy to clean, absence of separate toilet for male and female, toilet distance greater than 100 meters from the classroom and never wash hands before the meal was found to be the predictors of STHs.

The cumulative prevalence of STHs was 23.1%, with the most common prevalence for A. *lumbricoides* (11.2%) and T. *trichiura* (4.5%) in the current study. WHO has classified STH endemic areas as high transmission (where prevalence is >50%), moderate transmission (where prevalence is between 20%-50%), and low transmission (where prevalence is <20%) [1]. Based on the WHO classification of STHs infection prevalence, the study area would be classified into a moderate transmission category demanding the intervention of improved WASH. Also, this finding is far above from the short term national target reduction of the STH infections (1% V 23.1%) in Ethiopia. Thus, it is challenging to meet the national target of eliminating STHs infection by end of 2020 [26, 27]. This result is consistent with a study carried out in Butajira, South-Central Ethiopia (23.3%) [21]. The probable justification might be due to the correspondences of certain characteristics of schoolchildren and schools in the present study and studies mentioned above. These include the following factors: level of knowledge about the STHs, handwashing habits before a meal, toilet cleaning mechanisms, separate toilets for males and females. Also, it might be due to comparable study participants in these studies.

The prevalence of STHs in this study is higher than that of institution-based studies conducted in Were-abay (12.22%) and Babile town (13.8%) in Ethiopia [17, 28]. Similarly, an institution-based study conducted in Medebay Zana Woreda, North Western Tigray, Ethiopia showed a lower prevalence (12.7%) than the current study [23]. However, this result is lower than that of the studies conducted in Adwa town (69%) [29], Lumame town (54%) [30], Northern Gondar (66.7%) [6], Durbete town, Northwestern Ethiopia (54.9%) [22], and Zegie Peninsula (69.1%) [31]. This could be due to the fact that the topographic, sample size, study area, and period difference in which the population would improve their living standard, personal and environmental sanitation through time.

In this study, the prevalence of the skin-penetrating and orally-ingested STHs among the Schoolchildren were 6.8% and 16.34%, respectively. On the contrary to current finding, the study conducted in Argentina reported a higher prevalence of skin-penetrating STHs (37.5%) and a lower prevalence of orally-ingested STHs (9.8%) than our study [32]. This difference in prevalence might be due to variation in climate and soil type of the areas. Moreover, this difference might be due to the fact that Argentina’s study included rural study participants. However, this study included study participants from both rural and urban settings.

The prevalence of mixed infection with *A*. *lumbricoides*, *T*. *trichuira* and hookworm was 0.4% among the schoolchildren in this study. It is very low as compared to the findings of other studies conducted elsewhere [33, 34]. The differences in the prevalence of mixed infection among different study settings might be due to the presence of WASH intervention in some schools, and level of the knowledge, attitude and practice about the STHs.

Multivariable binary logistic regression analysis of predictors showed that odds of STHs were 2.03 times increased in schoolchildren living in large family size as compared to those living in small family size. This is similar to the study findings from the Tigray region and Addis Ababa of Ethiopia [23, 35]. This might be due to that the overcrowded family situation makes an increased opportunity for parasitic worm transmission. Also, parents/guardians may not have adequate time to care for their many children.

The odds of being infected by STH increased 3.33 times for children who did not have a separate toilet room for males and females in School than children have a separate room for males and females. The finding is in agreement with the studies conducted in Jimma zone North West Ethiopia and rural Southwest China [36, 37]. This might be because of the fact that all of the studied children were found in the developing country with poor infrastructure (lack of separate toilet for male and female) may discourage the utilization of toilets.

The toilet not easy to clean was positively associated with STHs. This is consistent with study findings from Gondar, North West Ethiopia, Durbete town, North-western Ethiopia, and Osun State, Nigeria [18, 22, 38]. This might be attributed to the fact that the toilet not easy to clean (not cemented, concrete, ceramic) may raise the risk of exposure to unhygienic conditions and leads to STHs infection. In addition, it might be explained by a lack of access to water for drinking and domestic purposes in the study area.

Multivariable analysis revealed that odds of STHs were 2.08 times higher in Schoolchildren who have inadequate knowledge as compared to those who have adequate knowledge about STHs. Similar findings reported from studies conducted in Wondo Genet district, Southern Ethiopia [39]. This might be attributed to the fact that schoolchildren who have good knowledge about the STHs are more likely to understand the mode of transmission and methods of prevention.

In addition, the odds of STHs were 3.82 times increased for children who had never washed their hands before a meal as compared to those who had always washed their hands. This finding is consistent with the studies done in Delgi school children, North Gondar Ethiopia, and rural schoolchildren in Abaye Sub district, Tigray regional state, Ethiopia [18, 28]. This might be due to a lack of awareness of children on how STHs are transmitted to humans. Besides, eating meals without washed hands enhances the faeco-oral transmission of STHs.

Moreover, multivariable analysis of predictors revealed that odds of being infected with STHs were 3.45 times increased for children who had travelled greater than 100 meters to access the toilet as compared to those who traveled 1–30 meters. This study finding is in line with studies conducted in North Gondar, Northwest Ethiopia and rural Communities in Honduras [6, 40]. This might be attributed to the fact that long toilet distance from classroom may discourage the utilization of toilets which inturns increase the tendency of open field defecation.

## Limitation of the study

The current study has several strengths. Among these, in the fact that we enrolled in a relatively large number of SAC (n = 1080) from multiple Schools and determined prevalence data for different STHs. Also, we made an effort to measure and accounted for a several potential confounders that can independently describe the association among the variables of interest. However, the following limitations should be taken into attention while inferring our findings. First, the present study was carried out among Schoolchildren living at a technology village of Hawassa University. Hence, the findings may not be generalizable to School-age children living at home or not enrolled in Schools. Second, the findings of this study might have been affected by reporting bias due to some part of the information was collected by the participants self-report. It is likely to have deliberate misreporting of behavioral related factors such as handwashing practice before eating a meal, handwashing practice after using the toilet, use of soap for hand washing, a habit of consuming raw vegetables and fruits, the practice of shoe wearing, a habit of nailbiting, and fingernail status (social desirability bias). Consequently, the magnitude of these predictors might have been underestimated and as such the association of these predictors with STHs might have been diminished. Third, like many other observational studies, we adjusted for several potential confounders using a multivariable logistic regression model. But, the residual confounding or confounding from unmeasured sources (e.g. lack of information about the home environment) cannot be excluded. Fourth, based on our descriptive finding, more than two-thirds (69.25%) of the study children received deworming treatment in the preceding 6 months of the survey. Hence, probable our study is expected to underestimate the underlying prevalence of the STHs. Finally, we might not capture the seasonal differences in the prevalence of STHs due to the cross-sectional nature of the study (i.e. children are only recruited within one month). Many previously conducted studies have witnessed that the prevalence of several STHs in the human population is subjected to inter-seasonal variability [41, 42].

## Conclusion

The prevalence of STHs reported in the current study is between the 20 to 50% range suggestive of moderate public health significance of STHs in a population. The highly prevalent species of STH infections were *A*. *lumbricoides* and *T*. *trichiura*. Also, concomitant infection with *A*. *lumbricoides*, *T*. *trichuira* and hookworm was identified. Moreover, the positive associations were identified between STHs infection and large family size, inadequate knowledge about the STHs, toilet not easy to clean, absence of separate toilet for male and female, toilet distance greater than 100 meters from the classroom and never wash hands before a meal. Reinforcing the existing fragile water, sanitation, and hygiene packages and regular deworming of schoolchildren may support to reduce the burden of STHs. Also, increasing modern contraceptive methods utilization to decrease family size is recommended. The result of toilet distance from classroom difference on the prevalence of the STH infections should be further investigated.

## Supporting information

S1 File (DOCX)

S2 File (DOCX)

S1 Dataset (SAV)
