# Supplementary material for: Prevalence of soil-transmitted helminthes and its association with water, sanitation, hygiene among schoolchildren and barriers for schools level prevention in technology villages of Hawassa University: Mixed design
Source: PLoS One. 2020 Sep 24;15(9):e0239557. doi: 10.1371/journal.pone.0239557 (PMC7514018; doi:10.1371/journal.pone.0239557)
Supplement: S1 File — (DOCX) [file pone.0239557.s001.docx]

# English version questionnaire

**Hawassa University**

**College of Medicine and Health Sciences**

**School of Public Health**

***Questionnaire designed to study the STH and its association with water, sanitation, hygiene (WASH) among Schoolchildren in technology village of Hawassa University, Southern Ethiopia***

**1. Information sheet**

Hello! My name is _------------------------------------------------------_. I am a data collector in a survey being conducted about STHs under the College of Medicine and Health Sciences of Hawassa University. The aim of this study is to assess the prevalence of STHs and its association with WASH among Schoolchildren in technology village of Hawassa University, Southern Ethiopia. The purpose of this study is to generate evidence about the STHs among Schoolchildren in technology village of Hawassa University. The study may help stakeholders, policy makers, responsible body, and others to take actions based on the finding. The study comprises various intimate and private questions, observation of School environment and taking stool sample. You are chosen to participate in this study by chance. Interview and stool sample collection will take not more than 30 minutes.

We assure you that there is no risk or harm in participation of this study. All information will be kept confidentially. Name of a participant will not be written or specified. Your privacy will also be protected and no one shall know your response.

This study benefits you that, you have the right to know your stool sample examination result. There is no incentive or payment for participating in this research. Likewise, findings of the study will show the magnitude and severity of the problem among study population. This in turn will help to design effective and appropriate measure for prevention and control of STHs.

You have full right whether or not to participate in this study. You may respond to all questions or you may not answer to the questions you don’t want to or you may quit your participation totally at any time you want. You can ask any questions which is not clear for you.

**2. Informed consent**

As to the information given ahead, participating in this study has no risk. In order to attain the objective of the study, your participation is vital. For this reason we are requesting your free will. You are selected randomly to participate in this study and your name will not be written on this form and the information you give will never be shared to others. Your genuine response to the interviews will be very important for the purpose of the study. You have a right to refuse in responding any question or the entire question at any time you want.

I have read this form or it has been read to me in the language I comprehend and understand all condition stated above.

Are you willing to participate in this study?

Yes □ No □

If “Yes" ...proceed with the interview.

If “No” ...........thank you and end.

Name of the principal investigator: Amanuel Yoseph

Cell phone Number - +251-915683578 E mail – amanuelyosep45@gmail.com

Name of interviewer___________________ Signature__________

Data of interviewer (Ethiopia calendar) _____/_____/_____

Result of interview: 1. Complete 2. Refused 3. Partially complete 4. Respondent not available

Cheeked by supervisor: Name______________ Signature_________ Data___/_____/____

| **Part I: Socioeconomic and Demographic characteristics** | | | |
| --- | --- | --- | --- |
| **S. No.** | **Question** | **Response** | **Remark** |
| 101 | School code |  |  |
| 102 | Grade level |  |  |
| 103 | Age |  |  |
| 104 | Religion | 1. Orthodox Christian 2. Protestant Christian 3. Muslim 4. Catholic 5. Others, (specify) __________ |  |
| 105 | Ethnicity | 1. Sidama 2. Amhara 3. Hadiya 4. Oromo 5. Wolayita 6. others |  |

| 106 | Mothers’ educational status | 1. Illiterate 2. Informal education (read and write) 3. Formal education _______ |  |
| --- | --- | --- | --- |
| 107 | Fathers’ educational status | 1. Illiterate 2. Informal education( read and write) 3. Formal education _______ |  |
| 108 | Mother’s occupation | 1. house wife 2. Self-employee 3. Merchant 4. Farmer |  |
| 109 | Father’s occupation | 1. Self-employee 2. Merchant 3. Government employee 4. Daily labourer 5. Others |  |
| 110 | Family size |  |  |

| **Part II. Water, sanitation and hygiene facility observation** | | | |
| --- | --- | --- | --- |
| S.No. | Question | Response | Remark |
| 201 | Does the school have water source?  (If answer no ask Q no. 301) | 1. Yes 2. No |  |
| 202 | If yes to Q no. 201, what is the current water source? | 1. Piped water _____ 2. Harvested (rain) ______ 3. Ground water (spring/well) _______ |  |
| 203 | Are there alternative sources nearby? | 1. Yes 2. No |  |
| 204 | If yes to Q no. 3, what types | 1. Surface water (lakes, ponds, streams and rivers) 2. Ground water (wells, spring) 3. Pipe water ______ |  |
| 205 | If the water is not for drinking, for what purpose do you use | 1. Laundary 2. Sanitation 3. Hygiene |  |
| 206 | How much drinking water is available per person per day? | 1. 1-2 liters 2. 3- 5 liters 3. > 5 liters |  |
| 207 | What is the daily/weekly frequency of the water supply? | 1. Daily 2. Every two days 3. Every three days 4. once a week |  |
| 208 | How much water is used for flushing toilets? | 1. No water for toilet 2. 1-5 liters 3. 5-10 liters 4. > 10 liters |  |

| **209** | Is the current water supply reliable? How long will it last? | 1. Two months 2. Six months 3. One year 4. longer than a year ________ |  |
| --- | --- | --- | --- |
| **210** | Are the water source protected from contamination?? | 1. Yes 2. No |  |
| **211** | If no to Q no. 210, what is the problem? | 1. Not Fenced around to protect cattle /animals 2. Leaky containers 3. No disinfection |  |
| **212** | Is treatment necessary?(school water ) | 1. Yes 2. No |  |
| **213** | Is treatment possible?(school water) | 1. Yes 2. No |  |
| **214** | If no, why |  |  |
| **215** | If yes, what type of treatment? |  |  |
|  |  |  |  |

| **Part III. Latrine observation** | | | |
| --- | --- | --- | --- |
| S. N | Question | Respone | Remark |
| 301 | Does the school have a toilet? (if no to go to q No. 401) | 1. Yes 2. No |  |
| 302 | If yes, how far is from the users? | 1. 10-30 meters 2. 31-70 meters 3. > 100 meters |  |
| 303 | What type of toilet is it? | 1. Trench latrine 2. Pit latrine 3. Water flushed latrine 4. Pour water latrine |  |
| 304 | Do boys and girls use separate toilet rooms?? | 1. Yes 2. No |  |
| 305 | If yes for Q no 304, what is the ratio of toilet to number of student per gender | 1. 1:25 female & 1:50 male 2. 1:100 female & 1:200male 3. >1:200 both |  |
| 306 | Are the toilets functional?  Functional (Fresh stool in the pits, smell and path to toilet) | 1. Yes 2. No |  |
| 307 | Is soap provided for toilet hand washing? | 1. Yes 2. No |  |
| 308 | Is the toilet easy to clean?  Easy (cemented, concrete, ceramic) | 1. Yes 2. No |  |

| Part IV. KAP of students towards STH prevention | | | |
| --- | --- | --- | --- |
| S.N | Question | Response | Remark |
|  | **Knowledge questions** |  |  |
| 401 | Do you have history of deworming pill in the preceding six months? | 1. Yes 2. No |  |
| 402 | Do you know soil-transmitted helminthes? | 1. Yes 2. No |  |
| 403 | If yes for q 402, which types of STHs? | 1. Ascariasis 2. Trichuriasis 3. Hookworm 4. Stroglodes |  |
| 404 | Which of the following is the cause of STHs? | 1.Bacteria  2. Virus  3. Parasitic worms  4. Fungus  5. I don’t know |  |
| 405 | Is STHs a transmissible disease/contagious? | 1. yes  2. No |  |
| 406 | If yes for question 405, what ways do you know about its transmission? (more than one answer is possible) | 1.By eggs that are passed in the faeces of infected people (Exposed excreta)  2. Consuming the vegetables and fruits that are not carefully cooked, washed or peeled  3. Eggs are ingested from contaminated water sources (water pollution)  4. Eggs are ingested by children who play in the contaminated soil and then put their hands in their mouths  5. Through penetrating skin  6. Others…………….  7. I don’t know |  |
| 407 | Do you know the symptoms of STHs? | 1. Diarrhea and abdominal pain  2. Loss of appetite  3. General malaise and weakness  4. Others…………………..  5. I don’t know |  |
| 408 | Is there a treatment for STHs? | 1. yes  2. No |  |
| 409 | Can STHs be prevented? | 1. yes  2. No |  |
| 410 | If yes to q 409, what methods do you know to prevent STHs? (more than one answer is possible) | 1. Hand washing with soap before meal and after toilet  2. By avoiding consumption of raw vegetables and fruits  3. Avoid playing with soil  4. Wearing shoes  5. By avoiding open field defecation  6. Avoid touching mouse with unwashed hands  7. By receiving a deworming tablet  8.Other, specify_______________  9. I don’t know |  |
| 411 | Which conditions increase the risk for STHs? | 1. Being preschool children  2. School-age children  3. women of reproductive age (including pregnant women in the second and third trimesters and breastfeeding women)  4. Adults in certain high-risk occupations such as tea-pickers or miners.  5. Others………………..  6. I don’t know |  |
|  | **Attitude questions** |  |  |
| 412 | Do you think STHs harm Schoolchildren? | 1. Yes  2. No |  |
| 413 | Do you think yourself at risk? | 1. Yes  2. No |  |
| 414 | If you take precautions, can the STHs infection be prevented? | 1. Yes  2. No |  |
| 415 | If you know that the raw vegetables and fruits are sources for the transmission of STHs, would you consume raw vegetables and fruits? | 1. Yes  2. No |  |
| 416 | Can STHs about infection be cured? | 1. Yes  2. No |  |
| 417 | Do you think the available information about STHs in School is sufficient? | 1. Yes  2. No |  |
| 418 | Do you think the elimination measures are sufficient for prevention of STHs in School? | 1. Yes  2. No |  |
| 419 | Do you think there is negative effect of infection on student academic performance? | 1. Yes  2. No |  |
| 420 | Do you think the government institutions able to eliminate the STH? | 1. Yes  2. No |  |
| 421 | If you have one of the symptoms of the disease do you go to the health facility? | 1. Yes  2. No |  |
| 422 | Do you think inappropriate latrine utilization can cause STH infection? | 1. Yes 2. No |  |
|  | **Practice questions** |  |  |
| 422 | Do you take safety precautions and prevention? | 1. Yes  2. No……………..Stop interview |  |
| 423 | If yes for q 422, which of the following precautions do you take to prevent STH infection? | 1. I wash hands with soap before meal and after toilet 2. I avoid touching the mouth with unwashed hands. 3. I avoid consuming raw vegetables and fruits. 4. I wear shoes 5. I avoid playing with soil 6. I avoid open field defecation 7. I receive deworming tablet 8. I keep my fingers clean by cutting nails 9. Others ……………………. 10. All of the above. |  |
| 425 | When do you wash your hands? | 1. Before meal and after meal 2. After toilet only 3. After toilet |  |
| 426 | How often do you wash hands before meal and after toilet? | 1. Always 2. Usually 3. Sometimes 4. Never |  |
| 427 | Do you use soap for washing hands? | 1. Yes 2. No |  |
| 428 | If no to Q no 427, why? | _____________ |  |
| 429 | If yes, When do you wash hands with soap | 1. Before meal and after meal 2. After toilet |  |

| **Part V : Laboratory finding** | | | |
| --- | --- | --- | --- |
| **S. No.** | **Laboratory information** | **Response** | **Remark** |
| 501 | Stool examination result  **if No to Q No 501 stop!** | 1. Ova/parasite seen 2. No ova /parasite seen |  |
| 502 | Identified parasite | 1. T .trichuria 2. A. lumbricoides 3. A. doudenale 4. E. vermicularis 5. Others specify |  |
| 503 | Type of infection | 1. Single infection 2. Double infection 3. Triple infection |  |
| 504 | Intensity of infection | 1. Low intensity 2. Moderate intensity 3. High intensity |  |

**In-depth interview Quide Questionnaire**

**Part I. Community health workers**

1. Would you explain about yourself (probe: professions and place of work)
2. Do you know about Soil transmitted helminthes (Ascariasis, Trichuris, and hook worm (If yes please meaning, cause, and complication)
3. What are important factors that can increase occurrence of STH in your vicinity?

(Hygiene problems, low latrine utilization and coverage, others)

1. What prevention strategies practice focused on STH in schools?

(Have you ever considered environmental measures like latrine utilization, hygiene promotion as prevention of STH?)

1. How do you rate Water, Sanitation, and Hygiene status of schools in your kebele?

Probe (Water access, utilization, sanitation and hygiene (water access, and latrine coverage and utilization)

1. What is/are barriers for STH prevention methods (probe barriers for latrine coverage and utilization, sludge management, personal hygiene, water access) in your villages
2. Anything else?

**Part II. SCHOOL HEADMASTER**

1. Would you explain about yourself (probe: professions and place of work)
2. Do you know about Soil transmitted Helminthes (Ascariais, Trichuris, and hook worm (If yes please meaning, cause, and complication)
3. Do you know how a person gets infected with STH (what is/are way for transmission of STH?
4. What are important factors that can increase occurrence of STH in your school?

(Probe, Hygiene problems, low latrine utilization and coverage, others)

1. Do you know how you can prevent yourself from getting diseases?
2. What prevention strategies practice focused on STH in your school?

(Have you ever considered environmental measures like latrine utilization, hygiene promotion as prevention of STH?

1. How do you rate Water, Sanitation, and Hygiene status of your school?

Probe (Water access, utilization, sanitation and hygiene, latrine coverage and utilization)

1. Is there ways that promotes hand washing practices of students in your school?
2. Anything else?

**Part III. Students**

1. Would you explain about yourself? (probe name, grade level and other roles)
2. Do you know about Soil transmitted Helminthes (Ascariais, Trichuris, and hook worm (If yes please meaning, cause, and complication)
3. Do you know how a person gets infected with STH (what is/are way for transmission of STH?
4. How do you rate Water, Sanitation, and Hygiene status of the kebeles particularly the schools? Probe (Water access, utilization, sanitation and hygiene (face washing, and latrine coverage and utilization)
5. What is/are barriers for school STH prevention methods (probe barriers for hand washing habits, antibiotic utilization, latrine coverage and utilization) in your schools?
6. Anything else?
