# Supplementary material for: Prevalence of soil-transmitted helminthes and its association with water, sanitation, hygiene among schoolchildren and barriers for schools level prevention in technology villages of Hawassa University: Mixed design
Source: PLoS One. 2020 Sep 24;15(9):e0239557. doi: 10.1371/journal.pone.0239557 (PMC7514018; doi:10.1371/journal.pone.0239557)
Supplement: S2 File — (DOCX) [file pone.0239557.s002.docx]

Sidaamu Afii Xa’muwa

Xa’mmuwa konni woroonni antanooti illaachishshsanohu egenno, rosischona, mageeshi dhibbinooro xawissanno ka’iimishiniiti, kunino saise doorantino sidaamu zoone woradana giddoyaanno Hawasssi gangawi woradira, borrichu woradirana, wondogeneetete woraadda giddo illete burdichchi (1-9) dirrinoo qaaquliraati.

Keereho; su’ma’ya yinanni. Hawaasi Yuniversite fayyimmate koleejenitti dawwomohu/maahuikkanna ki’ne doorantinooni manni leddo taje gambaassamaoraati dawoomahu/mohu. Taje gamba asseemo/ma wooyitteno ayimmakki/su’maaki bade leelishanoricho diboreessinaanni.

Sidaamu zoonera sasu woradi giddo loosaninoo xiinxallora . Xiinxxalo illachishahano hu bushshuni taraabbano da’muulla qaru korkaatimaatiro bade afatedaafiraati .Taje gamba aseemmo woyiteno ayimmakki bade leelishano richchodi boreessinani. Xa’meena kulattoereno wolu mannira horontani dikuleemmo. Aante noo xa’muwara gunni .Tenne xa’muwara dawarattonke/tanke daafira lowo geeshsha jajateni galaxeemmo. Ane ledo harunsate mitteenni ikkattoe/tae? Xa’muwa xa’matenna dawarate 40 daqiiqa calla adhano. Danchcha fajjooshshekkii kkiro hanafa dandiineemmo. Hananfo?

*Worada ollaa rosu mine barra / /*

*Taje gamba asanohu su’ma coode*

| **Gaamo I: dagatenamiinjuakatalainohuni** | | | |
| --- | --- | --- | --- |
| **A.K** | **Xa’muwa** | **akata** | **qaagiishsha** |
| 101 | Rosu mini coode |  |  |
| 102 | Rosu deerra |  |  |
| 103 | Diro |  |  |
| 104 | Ama’naki hiiteneeti? | 1. Ortodoksete 2. Protestaantete 3. Isilaamaho 4. Cathoolikete 5. Konini wole: xawisi |  |
| 105 | Hiite daga giddo gamamatto/ta? | 1. Sidaama 2. Amaara 3. Hadiya 4. Oromo 5. Wolaita 6. Konini wole: xawisi |  |

| 106 | Amate rosu deerra? | 1. Dirossinote/ho 2. Borreesanana nabbawa dandiitanote/ho 3. rosorossinote |  |
| --- | --- | --- | --- |
| 107 | Annu rosu deerra? | 1. Dirossinote/ho 2. borreesanana nabbawa dandiitanote/ho 3. roso rossinote/ho |  |
| 108 | Amate loosi maati? | 1. minaamaate   1. Baatto loosidhe galtano 2. daddalaanchote 3. barru loso losannote 4. wole(xawisi) |  |
| 109 | Annu loosi maati | 1. Baatto loosiregalanoho 2. daddalaanchoho 3. mangistete looso 4. barru loso losano 5. Konini wole: xawisi |  |
| 110 | Minu maate kirro me’ete? |  |  |

| **GaamoII. Wayi, keeraanchchimanna rosu mini wayi amado laino xa’mo** | | | | | | |
| --- | --- | --- | --- | --- | --- | --- |
| **A.K** | **Xa’muwa** | **qolo** | | | | **qaagiishaha** |
| **201** | Rosi’ne mini wayi nooho?  (**dawaro dino ikituro xammo 301 sa’i**) | 1. Ee 2. Dinoosiho | | | |  |
| **202** | Noosiha ikkiro xahiikiini afirano? | 1. Buuambuni 2. Xeenu waaqoqombe 3. Baattote gido fulano waa | | | |  |
| **203** | Rosu mini qooxeesira waa afi’naniti wole doogo no? | 1. Ee 2. Dino | | | |  |
| **204** | Aliidi xa’mora qoloki “ee” ikkituro mayi dani waa afi’nani? | 1. Daadano/garinowaa(garbuwaa, koofichuwaa,gidaawow.k.l.) 2. Baattote gido fulanowaa(bichcho,umoyi bale) 3. buuambuwaa | | | |  |
| **205** | Agate  horonsi’nayikiikkiromaahohoronsinani? | 1. Hayishirate(udunne ) 2. Keeraanchimate agarooshira 3. Umu umu keeraanchchimmara | | | |  |
| **206** | Mittu manchi barruni angani waa mageeshsha afirano? | 1. 1-2 leetire 2. 3- 5 leetire 3. > 5 leetire | | | |  |
| **207** | Barruni/lamalateniwaame’e hingeafi’nani? | 1. Barrutuqa 2. Layinkilayinkibarrini 3. Sayikkisayikkibarra 4. Lamalatenimitte hinge | | | |  |
| **208** | Shumate mine hayishshate mageeshshi waa | 1. Hattoo korkaatira | horonsi’nani | wayi | dino. |  |
|  | horonsi’nani? |  |  |  |  |  |
|  |  | 2. 1-5 leetiregeeshsha |  |  |  |  |
|  |  | 3. 5-10 lleetiregeeshsha |  |  |  |  |
|  |  | 4. >10 leetire |  |  |  |  |

| **209** | Xaa horonsi’dhinani wayi abbine ka’neenna mageeshsha keeshshano? | 1. lame agana 2. 6 agana 3. 1 diro 4. 1 dirialeeni |  |
| --- | --- | --- | --- |
| **210** | Waa afi’naniwi agaraminoho/ huxxaminoho? | 1. Ee 2. Dee’ni |  |
| **211** | Agaraminokiha ikkiroqarrumaati? | 1. Saada eanoki gede dihuxxaminoho 2. Kuusamino wini cocoano waati 3. xaginsoonikkiho |  |
| **212** | Waa xagisa hasiissano**?(rosu mini wayi)** | 1. Ee 2. Dee’ni |  |
| **213** | Waa Xagisa dandiinani? | 1. Ee 2. Didandiinanni |  |
| **214** | Dandiinanikki ikkiro mayira? |  |  |
| **215** | Dandiinaniha ikkiro hiittooni xaginsani |  |  |
|  |  |  |  |

| **Gaamo III. Shumate mine laiyoohumni** | | | |
| --- | --- | --- | --- |
| **A.K** | **Xa’muwa** | **dawaro** | **qagiishsha** |
| 301 | Rosu mini giddo shumate mini no  (**dawaro dino ikiro xamo 401 hanafi)** | 1. Ee 2. Dino |  |
| 302 | Nooha ikkiro horonsirano manniwini mageeshsha fafano? | 1. 10-30 meetire 2. 30-70 meetire 3. > 100meetire |  |
| 303 | Shumate mini hiittooho? | 1. Gadawa Ummooni baleeti 2. Qoxesaho afanni haquni lonsonnite 3. Waa ledo loonsoyite 4. Horonsi’neka’ne waa dunnannite |  |
| 304 | Seennunna labbalu babbaxitinowa horonsidhano? | 1. Ee 2. Dee’ni |  |
| 305 | Babbaxitinowa horonsidhanohaikkiro mitte hige magee labbali/seeni horonsirano | 1. 1:25 seeneho& 1:50labbaloho 2. 1:100 seeneho& 1:200 labbaloho 3. >1:200 lamunkura |  |
| 306 | Shumate mini xa horonsi’naniho | 1. Ee 2. Dee’ni |  |
| 307 | Shumate mine anga hayishi’nani gede saamunu  noosiho? | 1. Ee 2. Dino |  |
| 308 | Shumate mini feyaate injaanoho? | 1. Ee 2. Dee’ni |  |

| **Gaamo IV. Bushshuni taraabbano godowu da’muula gargadhate Egenno, rosichchonna wo’naalsha lainohuni** | | | | |
| --- | --- | --- | --- | --- |
| **A. K** | **Xa’muwa** | **dawaro** | **qagiishsha** | |
|  | **Egenote xamo** |  |  | |
| 401 | Saa’u barra gido godowu taraabbano aka’me kinnine hadhito? | 1. Ee 2. Di hadhomo |  | |
| 402 | Bushshu widooni taraabbano godowu da’muula maatiro  afootto/ta? | 1. Ee 2. Diafoommo/mma |  | |
| 403 | Xamo 402 ee yitoro/yotara hiko | 1. Asikariasis 2. Tikurayasis 3. Bushu giddo damulucho 4. Istergilosayasi |  | |
| 404 | Bushshu widooni taraabbano godowu da’muula malatisa mati? | 1.Baakeria  2. Wayires  3. Bushu gido damula  4. Fanigese  5. Diaffoma |  | |
| 405 | Kuni bushshu widooni dhibbi manchuni mancho taraawanno yite heedaato/ta? | 1. Ee  2. de’ni |  | |
| 406 | 405kki xa’mora dawaarokki ee yitoro/taro hittonni tarawaano yite heedaata/to?(mituni aleenni dorra dandinanni) | 1. Shumate widoni fultano damulani  2. Bura sagale itateni.  3. Dafamino waa aganteni.  4. Biso xororise  5. Dafamino bushini  6. Biso xororse  7. balunku dawaroote  8. Wole xawisi  6. diaffoma/ma |  | |
| 407 | Bushshu widooni taraabbano godowu malaata affaato? | 1. Duena godowa gama 2. Sagale giwisa 3. Dafurasa and wolqa huna 4. Wole xawisi 5. Diafomo |  | |
| 408 | Bushshu widooni taraabbano godowu dhibbira kitibaatu noosi? | 1. Ee  2. de’ni |  | |
| 409 | Bushshu widooni taraabbano godowu dhibbi amadikkinni balanxe gargadha dandinanni? | 1 ee  2. de’ni |  | |
|  |  |  |  | |
| 410 | 405kki xa’mora dawaarokki ee yitoro/taro hittonni tarawaano yite heedaata/to?(mituni aleenni dorra dandinanni) |  |  | |
|  |  |  |  | |
| 404 | Sagale itate albaanina shumate mine horonsidhe kae angakki me’e hige hayishiratto/tta? | 1. ganynyine 2. lowo yanna 3. Saesae 4. horonta |  |  |
| 405 | Anga hayishirate saamuna horonsiratto/tta | 1. Ee 2. Dee’ni |  | |
| 406 | Aliidi xa’mora dawaroki dee’ni ikkituro mayiira? |  |  | |
| 407 | Horonsiratto/tta ikkiro mamoote? | 1. Sagale ita’ya albaaninna itekae 2. Shumate minini fuleema/mo wote |  | |
| 408 | Bushshuni taraabbano godowu da’muullara qaru qaru korkaati maati? | 1. Cilho mula darga ofolate 2. Wayi gawajjo 3. Ayarete gawajjo |  | |
| 409 | Shumate mine garunni horonsira hooga bushshuni taraabbano godowu  da’muullara sayisse uyitanoha lawaahe? | 1. Ee 2. Dilawanoe |  | |
| 410 | Aliidi xa’mora dawaroki ee ikkituro hiitto? | 1. Shumate minini dange anga hayishira hoogateni 2. Tareessitano lubbuwani (lawisha tennuni) |  | |
| 411 | Shumate mine garunni horonsirateni hittooni  bushshu widooni taraabbano godowu da’muula gargadhineemo? | 1. Mulawa darga shumate ofola hoogateni 2. Tareessitano lubbuwa gargadhahoogateni 3. Sagale itate albaaninna gedensaani, shumate mine   horonsi’ne ke’ne anga hayishira hoogateni   1. Qinsheemo wote anga hayishirateni |  | |

| A.K | XA’MUWA | Dorranni doorsha. | kode |
| --- | --- | --- | --- |
| 411 | Hiko dani Bushshu widooni taraabbano godowu dhibbira | 1. Daima ikka 2. Rosu mini qaquli 3. Ammuwo (godowuni norinal qansitano amuwi) 4. Jajabu mani reqeci assano loso losanori 5. Wole xawisi 6. Diafomo |  |
|  | Xibu nonsa laio kenani xamo |  |  |
| 412 | Kunni bushshu widooni taraabbano godowu dhibbi lowontanni hantalaamoho/ gawaajanno) yite hedaato/ta? | 1. ee  2. de’ni |  |
| 413 | Ati umikinni bushshu widooni taraabbano godowu dhibbira reqqecci yototta/ta affooto/ta? | 1. ee  2. de’ni |  |
| 414 | Bushshu widooni taraabbano godowu dhibbinni daffamatewinni balanxe qorophiniro gantanni yite hedaato/ta? | 1. ee  2.de’ni |  |
| 415 | Bushshu widooni taraabbano godowu dhibbi tarawoora kaimu sadaate yiniro,maala woy ado saga’lato/ta? | 1. ee  2. de’ni |  |
| 416 | Bushshu widooni taraabbano godowu dhibbinni hurranni yite hedaato/to? | 1. ee  2. de’ni |  |
| 417 | Bushshu widooni taraabbano godowu dhibbi dafira gobbankera uyinonniti ikkado tajeeti yite hedaato/ta? | 1 ee  2. de’ni |  |
| 418 | Konne taraawanno dhibba mangitete urrinshsha gargara danditanno yite hedaato/ta? | 1. ee  2. de’ni |  |
| 419 | Rosanote rose gumi Bushshu widooni taraabbano godowu dhibbinni gawaajamanno yite hedaato/ta? | 1 ee  2. de’ni |  |
| 420 | Konne taraawanno dhibba mangitete urrinshsha gargara danditanno yite hedaato/ta? | 1. ee  2. de’ni |  |
| 421 | Kooronu dhibbi malaati mitu naffa lelihero hakkimete minira ha’raato/ta? | 1. ee  2. de’ni |  |
| 422 | Shumate mine garuni horosira hogiro bushshu widooni taraabbano godowu dhibira reqechi assano. | 1. ee  2. de’ni |  |
|  | **DHIBBIRA DAGOOMU AFIRINO ROSIICHCHO KENAANNI XA’MUWWA** |  |  |
| 423 | Bushshu widooni taraabbano godowu dhibbi taraawo gargadhaate adhoto/ta qorophpho hitteneeti? | 1. Anga samununi hayiremo shumate mine gedensana sagale itate albani 2. Hayishantinoki angani afo diamadhemo 3. Bura saga dietemo 4. Kowate wodhemo 5. Busha digodolemo 6. Damulate kinine adhemo 7. Qubeya harasiremo 8. Others 9. Balanku dawarote |  |

| **gaamo V : Laboratorte buuxiguma** | | | |
| --- | --- | --- | --- |
| **A.k** | **Labratoorete buuxo** | **dawaro** |  |
| 501 | Shumate mirmare/buxo  **Hogoru urisi** | 1. Damula no 2. Damula dino |  |
| 502 | Bandoni damula | 1. Tirakuur 2. Asikarise 3. Bushu gido damulcho 4. Isirgilodise 5. Wole |  |
| 503 | Damulate dana | 1. Mitee 2. Lamee 3. Sase |  |
| 504 | Damulate batiyne | 1. Lowo gesha shima 2. Mererima gesha shima 3. Batiyne damula |  |

**Galaxxemmohe!!!**
